# Impact of 6 months' Use of Intermittently Scanned Continuous Glucose Monitoring on Habitual Sleep Patterns and Sleep Quality in Adolescents and Young Adults with Type 1 Diabetes and High-Risk HbA1c

**DOI:** 10.1155/2023/1842008

**Published:** 2023-03-28

**Authors:** Shelley Rose, Barbara C. Galland, Sara E. Styles, Esko J. Wiltshire, James Stanley, Martin I. de Bock, Paul A. Tomlinson, Jenny A. Rayns, Benjamin J. Wheeler

**Affiliations:** ^1^Department of Women's and Children's Health, Dunedin School of Medicine, University of Otago, Dunedin, New Zealand; ^2^Department of Pediatrics and Child Health, University of Otago Wellington, Wellington, New Zealand; ^3^Department of Human Nutrition, University of Otago, Dunedin, New Zealand; ^4^Pediatric Department, Te Whatu Ora Capital, Coast and Hutt Valley, Wellington, New Zealand; ^5^Biostatistical Group, Dean's Department, University of Otago Wellington, Wellington, New Zealand; ^6^Department of Pediatrics, University of Otago, Christchurch, New Zealand; ^7^Pediatric Department, Te Whatu Ora Waitaha Canterbury, Christchurch, New Zealand; ^8^Pediatric Department, Te Whatu Ora Southern, Invercargill, New Zealand; ^9^Endocrinology Department, Te Whatu Ora Southern, Dunedin, New Zealand; ^10^Pediatric Department, Te Whatu Ora Southern, Dunedin, New Zealand

## Abstract

**Background:**

The bidirectional relationship between sleep and blood glucose levels may particularly affect adolescents and young adults (AYA), who are more likely to experience less healthy glycemic outcomes and more disrupted sleep patterns. To date, few data exist describing the impact of intermittently scanned continuous glucose monitoring (isCGM) on habitual sleep patterns and sleep quality in AYA with type 1 diabetes (T1D).

**Objective:**

To evaluate the impact of 6-month use of isCGM on habitual sleep and wake timing, sleep duration, frequency, and duration of night-time awakenings, sleep efficiency, and perceived sleep quality in young people with T1D and HbA1c ≥ 75 mmol/mol. *Participants.* The study recruited 64 participants aged 13–20 years (mean 16.6 ± 2.1), 48% female, diabetes duration 7.5 ± 3.8 years, 41% Māori or Pasifika, and a mean HbA1c 96.0 ± 18.0 mmol/mol [10.9 ± 3.8%]; 33 were allocated to an isCGM plus self-monitoring blood glucose [SMBG] intervention, and 31 were allocated to the SMBG control group.

**Methods:**

Participants completed 7-day actigraphy measures and the Pittsburgh Sleep Quality Index questionnaire at the baseline and at 6 months. Regression analyses were used to model between-group comparisons, adjusted for baseline sleep measures.

**Results:**

At 6 months, subjective measures for overall sleep quality, latency, duration, efficiency, night-time disturbances, use of sleep medications, and daytime dysfunction were similar between the groups. Regression analyses of actigraphy found no significant differences in objectively measured sleep timing and duration across the week after adjusting for age, the period of the school year, and baseline sleep values.

**Conclusions:**

The use of first-generation isCGM in addition to finger-prick testing did not impact objective or subjective sleep measures in AYA with T1D, elevated HbA1c, and highly variable sleep patterns. Research using alternative interventions for improving glycemic outcomes and habitual sleep-wake timing, duration, and perceived sleep quality is warranted in this population group.

## 1. Introduction

Sleep and blood glucose levels in type 1 diabetes (T1D) likely have a bidirectional relationship, where glycemic variation and suboptimal glucose control may impact habitual sleep-wake timing and sleep quality, and unhealthy sleep patterns negatively impact diabetes management [1–4]. Adolescents and young adults (AYA) are at a developmental stage where changes in their circadian rhythm can result in a shift in habitual sleep timing, and the natural drive to fall asleep may occur later than in younger children [5]. A consensus report from the National Sleep Foundation (NSF) in the U.S. provides age-specific sleep recommendations of 9–11 hours for 13-year olds, 8–10 hours for 14–17-year olds, and 7–9 hours of sleep each night for young adults [6]. However, the onset of puberty in adolescence contributes to a shift in circadian rhythm and a phase delay in sleep onset and offset timing. Other factors, including after-school activities or employment, completing homework, and the time spent using devices and following social media, may also impact when young people sleep at night [7]. As a result, many AYA accumulate a sleep deficit across the week, requiring recovery with longer sleep duration on weekends. Furthermore, at a time when the biological factors impacting sleep regulation are more likely to cause disrupted sleep patterns [7, 8], AYA with T1D are more likely to be entering a phase of worsening glycemic outcomes [9], reaching an HbA1c range that would be considered ‘highrisk” for developing diabetes-related complications (>75 mmol/mol [≥9%]) [10, 11]. Highly variable glucose levels may negatively impact sleep duration and quality [12, 13], and a disrupted sleep-wake cycle can lead to daytime sleepiness, inactivity, and appetite dysregulation [1, 14], all of which may impact behavioral aspects of diabetes management and difficulties achieving glycemic targets [3, 15].

Several factors can impact sleep health in young people with T1D. Overnight glucose fluctuations have been associated with changes in sleep architecture and increased sleep disturbance [16, 17], and studies using objectively measured sleep in AYA have linked difficulties self-managing T1D and poorer glycemic outcomes with shorter sleep duration and worse sleep quality [18]. Furthermore, we have previously demonstrated poorer sleep quality and later sleep onset times in AYA with highly elevated HbA1c (96.0 ± 18.0 mmol/mol [10.9 ± 3.8%]) compared to controls without diabetes [19].

Evidence now exists to support the use of continuous glucose monitoring (CGM) technology, including intermittently scanned CGM (isCGM) and real-time CGM (rtCGM), as an effective tool in improving glucose time-in-range (TIR) and HbA1c compared with self-monitored blood glucose (SMBG) assessment in children and young people with T1D [20–23]. The isCGM system measures interstitial glucose every minute but requires the sensor to be scanned with a reader or smartphone app every 8 hours to capture all the data. The 14-day glucose profile can be interpreted along the idea target range of 3.9–10.0 mmol/L (70–180 mg/dL) at least 70% of the time, which correlates with an HbA1c of 53 mmol/mol (7.0%) and less than 4% time below the target range (TBR) of <3.9 mmol/L (<70 mg/dL) [24]. Regular use of isCGM can lead to improvements in HbA1c and glucose TIR in children and adolescents over the short term [20] and has the potential to impact habitual sleep patterns and sleep quality positively [25]. However, few objectively or subjectively measured data exist to describe the impact of isCGM on sleep timing, duration, or quality in AYA with T1D.

The overall aim of this randomized controlled trial (RCT) substudy was to evaluate the impact of 6 months' unblinded first-generation isCGM use by assessing changes in habitual sleep and wake timing, sleep duration, and sleep quality in AYA with T1D and HbA1c well above-target. In this study, we measured sleep onset and offset timing, sleep duration, disturbances (frequency and duration of awakenings after sleep onset), sleep efficiency, and changes in sleep quality before and after 6 months of isCGM compared with SMBG. We hypothesized that participants using isCGM would, via multiple pathways (improved TIR; reduced diabetes and psychosocial burden), experience less variable sleep and wake timing patterns, improvements in sleep duration, and healthier sleep quality scores compared to those using SMBG at 6 months.

## 2. Methods

### 2.1. Participants

We recruited 64 AYA (age 13–20 years, inclusive) with established T1D (at least 12 months' duration) from three regional diabetes centers in New Zealand between April 2018 and November 2019 for a 6-month RCT comparing first-generation isCGM with SMBG (via finger-pricking) in addition to usual care, see the previously published full protocol [26]. Eligible participants had a preenrolment mean HbA1c ≥ 75 mmol/mol (≥9%) in the previous six months, requiring ≥0.5 units insulin/kg/day and no use of isCGM or rtCGM within the previous 4 months [26]. Written informed consent and assent were obtained from all eligible AYA before the baseline visit; additionally, consent was obtained from the parents of participants aged 15 years and under.

### 2.2. Study Design

**Figure 1:**
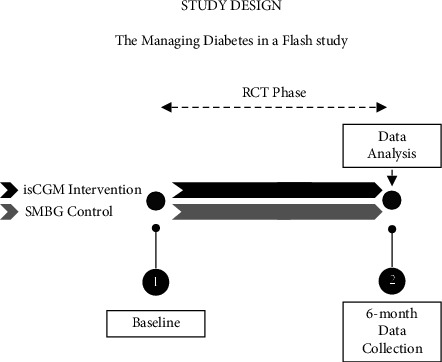
Managing diabetes in a flash 6-month RCT study design. At baseline, participants were randomly allocated to either the isCGM intervention (black arrow) using unblinded freestyle ibre to measure glucose levels with SMBG performed as needed (e.g., to confirm glucose level when measurements did not match clinical symptoms) or to the SMBG control group performing finger-prick tests only (gray arrow). Sleep assessments were completed at the baseline and 6-month data collection points; actigraphs were provided and worn a week prior, and the Pittsburgh sleep quality index questionnaire was completed at the baseline and 6-month data collection points, respectively.

This project is a substudy of a larger program investigating the impact of isCGM on glucose levels that included a 6-month RCT followed by a 6-month extension study where participants were randomly allocated at the baseline to either the 6-month isCGM intervention group (FreeStyle Libre 1 system, Abbott Diabetes Care, Witney, Oxon, UK) or the 6-month waitlist SMBG control group. When this study commenced, we used unblinded isCGM as the Freestyle Libre Pro system (blinded) was unavailable in New Zealand. In this substudy, participants wore an Actigraph device for one week prior to the baseline and 6-month assessments, completing the Pittsburgh Sleep Quality Index (PSQI) questionnaire at those visits (Figure 1).

In the post-RCT phase of this study, the SMBG control group switched to the isCGM intervention, and both groups used isCGM for the next 6 months to assess the impact on glycemic outcomes, but sleep assessments were not performed beyond the 6-month RCT phase (see the primary outcomes [27] and additional 6-month extension study outcomes [28] for more detail). The *Managing Diabetes in a Flash* study protocol was approved by the Ngāi Tahu Research Consultation Committee prior to obtaining approval by the New Zealand Health and Disability Ethics Committee (Ethics ref 17/STH/240). The trial was registered with the Australian New Zealand Clinical Trials Registry (ACTRN12618000320257p) after obtaining a Universal Trial Number U1111-1205-5784.

### 2.3. Sample Size Calculation

The study power is based on the primary outcomes of the RCT, where 90% of the participants (*n* = 58) would detect differences with 80% power in sleep measurement means (via actigraphy) between the intervention and control arms of approximately 0.75 SDs (a moderate-large effect size) and significant at the 0.05 level.

### 2.4. Demographic and Clinical Information

The following demographic data were collected: gender, age, prioritized ethnicity, [29] education, and employment status. The established index of geographical deprivation (NZDep13) was used to categorize socioeconomic status [30]. Clinical data, including the date of diagnosis with T1D, current insulin regimen, episodes of acute complications in the prior 6 months, comorbidities, HbA1c, and auxology, were collected from participants' electronic medical records. HbA1c was measured using a point-of-care (POC) analyzer (DCA Vantage Analyzer, Siemens Healthcare Diagnostics, Ireland) linked directly to the DCCT method via the National Glycohemoglobin Standardization Program. A report on average blood glucose (BG) levels and BG test frequency over the previous two weeks was obtained by downloading the participants' glucometer (CareSENS dual; i-SENS Inc., Seoul, Korea) using SmartLog software (SmartLog Diabetes Management Software, version 2.4.4, i-SENS Inc., Seoul, Korea). All data were collected and managed using REDCap, hosted by the University of Otago [31].

### 2.5. Sleep Assessments

#### 2.5.1. Actigraphy

All participants were asked to wear an accelerometer device (ActiGraph© wGT3X-BT, ActiGraph Activity Monitor Devices, RRID:SCR_008399) continuously (for seven days and nights) on their nondominant wrist. This enabled the objective assessment of habitual sleep onset and offset timing and the calculation of associated sleep variables. As reported in previous studies that objectively assess sleep in young people [25, 32], the ActiGraph devices were initialized to capture data every 15 seconds and MATLAB software (MATLAB, RRID:SCR_001622) was used to process outcomes using a count-scaled algorithm [33]. Taking the manually inserted timestamps indicating self-reported sleep and wake timing, the algorithm scanned from 2 hours prior to 3 hours following to measure sleep onset and offset objectively. Meltzer et al. [34] recommendations were followed to score sleep onset as the first 15 continuous minutes of sleep preceded by 5 minutes of wake time and sleep offset as the final 15 continuous minutes of sleep followed by 5 minutes of awake time.

Sleep variables were reported for weekdays (Sunday through Thursday) and weekends (Friday through Saturday), with at least 8 hours of wear time over a 24-h period required for a “valid day” of actigraphy. Participants with fewer than 2 valid days in total were excluded from the analysis. The weighted mean of sleep variables across all nights was calculated by weighting weekdays 5/7 and weekends 2/7. Actigraphy data were controlled for age and the period of the school year versus holiday or vacation time, given the known impact these have on sleep outcomes.

Objective sleep variables were defined as follows: the duration of time between sleep onset and offset (SPT, sleep period time); the SPT minus the duration of night-time awakenings (TST, total sleep time); sleep efficiency (SE, TST as a percentage of SPT); the number of awakenings (defined as ≥5 consecutive minutes awake); and the duration of time spent awake after sleep onset (WASO) across the SPT.

#### 2.5.2. Pittsburgh Sleep Quality Index

The 19-item Pittsburgh Sleep Quality Index (PSQI) [35] was used to subjectively measure participants' sleep timing and quality over the prior month. The PSQI was amended for this study to assess sleep in AYA under 19 years of age by removing two items relating to sleeping with a bed partner, as described in the study protocol [26]. With internal consistency (Cronbach's *α*) ranging from 0.71 to 0.73, the psychometric properties of the PSQI have been validated in studies involving different AYA groups [36–38], including a community sample of Australian teens aged 12–18 years showing internal consistency of 0.73 and subscale score correlations with the global score of 0.31 to 0.77 [36].

Seven domains for sleep quality are identified by the PSQI: subjective sleep quality, sleep latency, duration, sleep efficiency, disturbances, use of sleeping medication, and daytime dysfunction. These component scores (from 0 to 3) are summed to generate a PSQI global score (from 0–21), where >5 indicates poor sleep quality. In this study, the sleep duration scores were adjusted by +1 hour to reflect sleep recommendations for AYA <18 years [39].

### 2.6. Statistical Analysis

The demographic and clinical data are described as mean and standard deviation (SD) for continuous variables (age, diabetes duration, HbA1c, and BMI z-score) and the number and percent of participants for categorical variables (gender, ethnic group, NZDep13 group, and insulin regimen). The data were plotted against the normal distribution and assessed using the Kolmogorov–Smirnov test. Objective and subjective measures for sleep onset and offset were reported as mean (SD) for continuous variables or median (25^th^, 75^th^ percentile). To estimate the mean differences and 95% confidence intervals (CIs) between the isCGM intervention and SMBG control groups at 6 months, results were compared using analysis of covariance (ANCOVA) within a linear regression model, allowing for baseline values of the outcome as a covariate and adjusting for age and period of school year.

Histograms of each continuous variable and residual versus fitted plots from each linear regression were used to assess the suitability of sleep timing data for analysis. The number and duration of awakenings and sleep efficiency measured by actigraphy and the subjectively measured time to sleep onset were analyzed as log-transformed variables (SE was converted to 1–SE prior to log transformation). The log-transformed estimates were reported as ratios of the geometric means to compare the isCGM intervention relative to the SMBG controls.

The median, 25^th^, and 75^th^ percentiles for each PSQI sleep quality domain were described at the baseline and at 6 months, and the odds ratio and 95% CI for reporting a “poor score” at 6 months were calculated after adjusting for age. Statistical analyses were completed using Stata 16.1 (Statacorp, College Station, Texas, RRID:SCR_012763), and a two-sided*p* value of <0.05 was used to identify statistically significant differences between the two groups.

## 3. Results

### 3.1. Demographic and Clinical Information

The participants' baseline demographic data and clinical characteristics according to randomization are presented in Table 1. Thirty-three participants were allocated to the isCGM intervention group, and 31 participants continued using SMBG-only (the control group) for the 6-month RCT phase.

### 3.2. Actigraphy

An average of 6.0 and 5.2 days of actigraphy wear was captured for the isCGM intervention and SMBG control groups, respectively. Actigraphy data (≥2 valid days) from 23 participants in each group at 6 months were included in the analysis; 46 participants provided at least weekday actigraphy data at both the baseline and 6 months; however, only 37 participants provided weekend data at both time points, as described in Table 2. After controlling for age, period of the school year, and baseline sleep values, we found no substantial evidence for differences in objectively measured sleep variables between the groups, with the 95% confidence intervals across weekdays and weekends and the weighted mean suggesting that the mean difference between the groups could fall within a wide range (the mean difference column in Table 2).

### 3.3. Pittsburgh Sleep Quality Index (PSQI)

Tables 3 and 4 (baseline data as per Rose et al. [19]) describe the PSQI sleep quality scores from 33 participants in the isCGM intervention group and 31 participants in the control group at 6 months; all those in the SMBG control group provided subjectively measured sleep timing data, and 32 participants in the isCGM group. After controlling for age, the period of the school year, and baseline sleep values, we found little evidence for differences in sleep latency, wake time, time in bed, and sleep duration reported over the prior month. We also found no substantial evidence for a difference in the likelihood of reporting a “poor score” for each of the PSQI sleep quality domains after controlling for age. Participants in the isCGM group had a lower PSQI global score at both the baseline and after 6 months, but there was no difference between the groups at 6 months after adjusting for baseline values.

## 4. Discussion

The *Managing Diabetes in a Flash* study provided a unique opportunity to measure habitual sleep patterns and sleep quality in AYA with T1D and very high HbA1c and assess the impact of isCGM on sleep over a 6-month period. Prior research has shown that a complex and bidirectional relationship exists between sleep and glycemic control in T1D, where elevated blood glucose levels likely impact sleep and poorer sleep can negatively impact glycemia [1]. Emerging evidence has shown that AYA with T1D experience irregular sleep timing [18], duration [13], and sleep quality [12, 40], which may be associated with difficulties with diabetes self-management and suboptimal glycemic outcomes [1, 25]. By design, the participants in this study had HbA1c levels well above the target (96.0 ± 18.0 mmol/mol [10.9 ± 3.8%]). While both groups subsequently improved HbA1c and the isCGM arm showed improved diabetes treatment satisfaction [27] and aspects of quality of life [41, 42], both groups continued to show poor sleep patterns, and we found no evidence of a difference between the groups for objectively or subjectively measured sleep after 6 months of isCGM use in addition to routine care. This suggests that the addition of isCGM alone (despite some aspects of diabetes improving) does not improve these poor habitual sleep patterns and sleep quality in those who already face significant challenges trying to achieve recommended glycemic targets [43].

Our 6-month actigraphy data indicated highly variable sleep onset and offset occurred across the week (as evidenced by a high standard deviation from the mean for each variable) in both the isCGM intervention and SMBG control groups, which concurs with earlier reports linking sleep patterns with suboptimal glycemic outcomes in adolescents with T1D. [44, 45] Furthermore, sleep-wake timing among our participants indicated late sleep onset times (after midnight) across weekdays and weekends when compared to normative values for objectively measured nighttime sleep in adolescents [46]. Our observations concur with an earlier association between habitual sleep timing and above-target HbA1c in New Zealand children and adolescents with T1D, where sleep onset and offset were approximately 30 minutes later than in those with less healthy glycemic control [25].

Regression analysis of our actigraphy data found no strong evidence for suggesting differences in objectively measured sleep timing after controlling for age, the period of the school year, and baseline sleep values. The use of isCGM did not appear to impact sleep onset (MD-36 minutes (95% CI −76, 4) *p* = 0.078) or sleep offset times (MD-23 minutes (95% CI −69, 22); *p* = 0.307) after 6 months, with broad confidence intervals for each variable suggesting that a wider variation in the mean difference between the groups is possible. These results were not a total surprise, given that there was a nonsignificant difference in HbA1c between the groups at the end of the RCT phase, despite a 2.8 times higher frequency of glucose monitoring among those using isCGM [27]. It is plausible to suggest that these highly variable sleep patterns more broadly reflect the struggle some AYA have achieved for a consistent daily routine, which may be influenced by prebedtime activities (cultural or family activities, work or study, use of devices and social media, or diabetes-related tasks), ethnicity, or economic disadvantage that may impact habitual sleep patterns and sleep quality [7, 47].

Overall, 43% of the participants in this study reported sleep durations well short of recommendations for their age [6], with the addition of isCGM to routine care failing to make a difference to objectively measured total sleep time (MD-17 mins; *p*=0.353) and subjectively measured hours of sleep per night (MD-26 mins; *p*=0.230). Other studies in children and adolescents with T1D show an association between not getting enough sleep and the experience of daytime cognitive and behavioral difficulties [14, 48, 49]. Furthermore, Ohayon et al. [50] suggest that the following measures of sleep continuity are appropriate indicators of sleep quality at most ages: time to sleep onset ≤15 minutes, 1 or fewer night-time awakenings >5 minutes, wake after sleep onset ≤20 minutes, and sleep efficiency ≥85% [50]. Our estimate of sleep efficiency did not reflect increased sleep disturbances, and it is possible that our participants were attending to diabetes-related tasks in the evening before going to sleep and not having their sleep interrupted with rtCGM alarms, thereby accounting for less sleep disturbance overnight.

Overall, subjectively measured sleep outcomes in this study did not indicate that the use of isCGM made a difference in AYA sleep patterns or perception of sleep quality, although improvements in the PSQI global score were seen in both groups at 6 months. An earlier study by Al Hayek et al. [51] demonstrated improvements in PSQI sleep quality scores, an increase in SMBG frequency, and a decrease in HbA1c in young adults with T1D following 3 months' use of isCGM. Their results demonstrate the existence of a bidirectional relationship between sleep and glucose levels in young adults (mean age 20.9 years) who had a mean HbA1c (67.0 ± 10.9 mmol/mol [8.3 ± 1.0%]). However, the average age of our participants was lower (16.6 ± 2.1 years) and their mean HbA1c (96.0 ± 18.0 mol/mol) was much higher, and we were unable to replicate these benefits of isCGM on subjectively measured sleep variables. We believe that this demonstrates the strength of our study that examines the impact of isCGM on habitual sleep patterns in AYA who face significant challenges in achieving glucose levels in the target range and provides recommendations for overall sleep duration and quality.

Another key strength of this substudy is the ability to assess the impact of isCGM on habitual sleep patterns and sleep quality in an RCT study designed for AYA with highly elevated HbA1c levels. This allowed participant access to glucose monitoring technology that was not publicly funded and evaluated among ethnic groups (Māori and Pasifika) where uptake of diabetes technology is inequitable [52].

We used subjective sleep measures to assess sleep timing and quality; however, we did not screen for sleep disorders during the recruitment of participants, and this could have influenced the PSQI global scores at the baseline for some participants. Actigraphy data were captured with an average wear of 6.0 and 5.2 days for the isCGM and SMBG groups, respectively, and results from participants with ≥2 valid days were included in the analysis; however, for actigraphy-measured total sleep time, more than seven nights are ideal [53]. Missing actigraphy data could have influenced the reliability of some of the sleep measures. A further limitation is that we did not have access to blinded CGM data as the Freestyle Libre Pro system was not available in New Zealand at the study onset, and we did not conduct a subanalysis of sleep outcomes in the minority of participants using insulin pump devices. However, given this study population's very elevated HbA1c levels at the baseline and the primary endpoint showing a nonsignificant improvement in HbA1c at 6 months, it is perhaps unlikely that shorter-term changes in glucose time-in-range would have impacted habitual sleep patterns overall.

## 5. Conclusion

Adolescents and young adults with T1D and elevated HbA1c demonstrate highly variable sleep and wake timing, with late sleep onset times contributing to an overall sleep deficit. There were large variations in objectively measured sleep duration across the week, with many experiencing average amounts of nighttime sleep below recommendations for age. The use of isCGM in addition to SMBG was not sufficient to impact objective or subjective measures of sleep outcomes in young people with T1D, HbA1c levels well above-target, and highly variable sleep patterns. Research using alternative interventions for improving glycemic outcomes and habitual sleep-wake timing, duration, and perceived sleep quality is warranted in this population group.

## Figures and Tables

**Table 1:** Demographic and clinical characteristics of participants according to randomization.

Characteristics	Total (*n* = 64)	isCGM intervention group (*n* = 33)	SMBG control group (*n* = 31)
Age (years), mean ± SD	16.6 ± 2.1	16.5 ± 1.9	16.7 ± 2.2
Gender, *n* (%)			
Female	31 (48)	16 (48)	15 (48)
Male	33 (52)	17 (52)	16 (52)
Prioritized ethnicity, *n* (%)			
NZ European	37 (58)	18 (55)	19 (61)
Māori^a^	16 (25)	9 (27)	7 (23)
Pasifika	10 (16)	5 (15)	5 (16)
Asian	1 (2)	1 (3)	0 (0)
NZDep13^b^, *n* (%)			
Low deprivation (1–3)	19 (30)	10 (30)	9 (29)
Medium deprivation (4–7)	26 (41)	12 (36)	14 (45)
High deprivation (8–10)	19 (30)	11 (33)	8 (26)
Education/employment, *n* (%)			
In education (secondary)	42 (66)	21 (64)	21 (29)
In education (tertiary)	11 (17)	5 (15)	6 (19)
In employment	10 (16)	6 (18)	4 (13)
NEET^c^	1 (2)	1 (3)	0 (0)
BMI (*z*-score)^d^, mean ± SD	0.70 ± 1.00	0.67 ± 1.05	0.73 ± 0.96
Diabetes duration (yrs), mean ± SD	7.5 ± 3.8	7.0 ± 3.5	8.0 ± 4.0
Insulin therapy, *n* (%)			
MDI	55 (86)	29 (88)	26 (84)
CSII	9 (14)	4 (12)	5 (16)
Estimated TDD (units), median (IQR)	72 (31)	73 (33)	70 (24)
Mean units per kg	1.2	1.2	1.1
HbA1c (mmol/mol), mean ± SD	96.0 ± 18.0	94.0 ± 18.0	99.0 ± 18.0
HbA1c (%), mean ± SD	10.9 ± 3.8	10.8 ± 3.8	11.2 ± 3.8

NZ, New Zealand; BMI, body mass index; MDI, multiple daily injections; CSII, continuous subcutaneous insulin infusion; TDD, total daily dose; IQR, interquartile range; HbA1c, hemoglobin A1c. Preenrolment meanHbA1c ≥ 75 mmol/mol (≥9%) in the prior 6 months; HbA1c ranged from 68 to 130 mmol/mol at the baseline visit. ^a^Māori are the first inhabitants of Aotearoa NZ. ^b^NZ Dep13; marker of geographic deprivation. ^c^NEET; not in employment, education, or training. ^d^BMI (*z*-score); calculated using the Centre for Disease Control guidelines.

**Table 2:** Actigraphy sleep parameters for isCGM intervention and SMBG controls at the baseline and 6 months.

Sleep variables	*n*	isCGM intervention baseliine	*n*	SMBG controls baseline	*n*	isCGM intervention 6 months	*n*	SMBG controls 6 months	*n*	MD (95% CI) adjusted	*p*
Sleep onset (hh:mm)											
Weekdays	23	00:03 (1:38)	23	23:28 (1:00)	23	00:27 (1:31)	23	00:45 (1:05)	46	−29 (−72, 14)	0.179
Weekends	22	00:26 (2:14)	21	23:54 (1:19)	19	00:36 (1:48)	20	01:26 (1:31)	37	−58 (−122, 6)	0.073
Weighted mean^†^	23	00:09 (1:39)	23	23:35 (0:59)	23	00:31 (1:31)	23	00:54 (1:03)	46	−36 (−76, 4)	0.078
Sleep offset (hh:mm)											
Weekdays	23	07:48 (1:21)	23	07:48 (0:49)	23	07:48 (1:13)	23	08:03 (1:26)	46	−15 (−65, 34)	0.531
Weekends	22	09:00 (1:42)	21	08:37 (1:14)	19	08:29 (1:42)	20	09:02 (1:32)	37	−33 (−100, 35)	0.334
Weighted mean^†^	23	08:07 (1:18)	23	08:01 (0:42)	23	07:56 (1:11)	23	08:18 (1:16)	46	−23 (−69, 22)	0.307
SPT (hh:mm)											
Weekdays	23	8:05 (1:30)	23	8:18 (1:19)	23	7:20 (1:11)	23	7:19 (1:00)	46	−6 (−43, 31)	0.744
Weekends	22	8:09 (2:13)	21	8:43 (1:32)	19	7:53 (127)	20	7:35 (1:25)	37	16 (−37, 68)	0.546
Weighted mean^†^	23	8:23 (1:17)	23	8:24 (1:07)	23	7:25 (1:12)	23	7:24 (0:54)	46	5 (−40, 31)	0.798
TST (hh:mm)											
Weekdays	23	7:29 (1:24)	23	7:52 (1:22)	23	6:34 (1:17)	23	6:41 (0:54)	46	−13 (−52, 26)	0.518
Weekends	22	8:23 (2:07)	21	8:13 (1:47)	19	6:54 (1:28)	20	7:09 (1:44)	37	−18 (−74, 38)	0.521
Weighted mean^†^	23	7:44 (1:10)	23	7:57 (1:17)	23	6:37 (1:16)	23	6:49 (0:56)	46	−17 (−54, 20)	0.353
Awakenings (*n*)^†^											
Weekdays	23	1.0 (0.85)	23	0.6 (1.0)	23	0.8 (1.0)	23	0.8 (1.3)	46	1.0 (0.6, 1.7)	0.911
Weekends	22	1.0 (1.2)	21	0.5 (1.0)	19	1.0 (1.5)	20	0.8 (0.8)	37	1.4 (0.8, 2.3)	0.216
Weighted mean^†^	23	1.0 (0.8)	23	0.6 (1.1)	23	0.9 (1.1)	23	0.8 (1.1)	46	1.0 (0.6, 1.7)	0.882
WASO (mins)^††^											
Weekdays	23	34.5 (44.5)	23	17.2 (35.2)	23	23.3 (58.7)	23	32.1 (35.1)	46	1.0 (0.3, 3.0)	0.935
Weekends	22	32.1 (71.6)	21	8.9 (52.9)	19	54.0 (62.7)	20	16.7 (41.8)	37	3.7 (0.9, 15.2)	0.072
Weighted mean^†^	23	29.6 (40.6)	23	18.3 (45.3)	23	38.5 (62.7)	23	30.4 (42.8)	46	1.5 (0.6, 3.9)	0.428
SE (%)^††^											
Weekdays	23	93.5 (7.6)	23	96.2 (7.7)	23	94.3 (12.8)	23	93.7 (7.1)	46	1.1 (0.4, 3.0)	0.899
Weekends	22	93.6 (10.9)	21	98.4 (10.0)	19	88.1 (13.8)	20	96.4 (10.9)	37	0.3 (0.1, 1.2)	0.080
Weighted mean^†^	23	94.1 (7.0)	23	95.8 (7.5)	23	92.4 (13.3)	23	93.4 (9.6)	46	0.8 (0.3, 1.9)	0.546

isCGM, intermittently scanned continuous glucose monitoring; SBMG, self-monitoring blood glucose; SD, standard deviation; MD, mean difference; CI, confidence interval; SPT, sleep period time (elapsed time between sleep onset and offset); TST, total sleep time (time between sleep onset and offset, minus awakenings); awakenings, number of awakenings across the SPT; WASO, duration time awake after sleep onset; SE, sleep efficiency (TST/SPT^*∗*^100). Continuous variables presented as mean ± SD; linear regression model used to estimate MD (95% CI) between the groups at 6 months after adjustment for age, period of the school year, and baseline sleep values as covariates. ^†^The 7-day weighted mean of sleep variables across all nights was used in case of incomplete data across the seven days, where weekdays (Sunday to Thursday) were weighted 5/7, and weekends (Friday and Saturday) weighted 2/7. ^††^Awakenings, WASO, and SE data are presented as medians (IQR); MD (95% CI) between the groups at 6 months and log-transformed data expressed as ratios of geometric means for the isCGM intervention group relative to the SMBG controls after adjustment for age, period of the school year, and baseline values. In the case of SE, this was converted to 1-SE prior to log transformation).

**Table 3:** PSQI sleep timing in the isCGM intervention group and SMBG controls at the baseline and at 6 months.

Sleep timing	isCGM intervention baseline (*n* = 32)	SMBG controls baseline (*n* = 30)	isCGM intervention 6 months (*n* = 32)	SMBG controls 6 months (*n* = 31)	MD (95% CI)	*p*	MD (95% CI) after adjustment	*p*
Sleep latency (min)^†^	19 (10, 53)	30 (13, 50)	20 (10, 55)	20 (10, 40)	—	0.872	1.0 (0.7, 1.5)	0.941
Bedtime (hh:mm)	22:55 ± 1:29	22:57 ± 1:22	23:18 ± 1:34	22:51 ± 1:30	26 (−8, 61)	0.132	27 (−8, 63)	0.122
Wake time (hh:mm)	07:59 ± 1:57	07:45 ± 1:14	08:10 ± 1:37	08:02 ± 1:37	−2 (−51, 48)	0.949	5 (−44, 54)	0.848
Time in bed (hh:mm)	9:04 ± 1:48	8:48 ± 1:29	8:52 ± 1:24	9:11 ± 1:28	−31 (−72, 11)	0.149	−32 (−74, 11)	0.142
Sleep duration (hh:mm)	7:30 ± 1:32	7:08 ± 1:44	7:30 ± 1:20	7:49 ± 1:39	−24 (−67, 19)	0.216	−26 (−69, 17)	0.230

PSQI, Pittsburgh sleep quality index; isCGM, intermittently scanned continuous glucose monitoring; SBMG, self-monitoring blood glucose; SD, standard deviation; MD, mean difference; CI, confidence interval; sleep latency, time taken to fall asleep each night; bedtime, usual bedtime at night; wake time, usual time got up in the morning; time in bed; elapsed time between bedtime and wake time; sleep duration, actual hours slept at night. Continuous variables are presented as mean ± SD; *p* values for differences between isCGM intervention and SMBG controls are calculated using analysis of covariance to estimate MD (95% CI). The *p* values for differences between isCGM intervention and SMBG controls were estimated using linear regression with 95% CI after adjustment for age and period of school year, with baseline value as a covariate. ^†^Sleep latency data presented as median (25^th^, 75^th^ percentiles); *p* value for estimated MD (95% CI) in sleep latency at 6 months assessed using the Mann−Whitney *U* test; MD (95% CI) after adjustment of log-transformed data expressed as ratios of geometric means.

**Table 4:** PSQI sleep quality in the isCGM intervention group and SMBG controls at the baseline and at 6 months.

Sleep quality	isCGM intervention baseline (*n* = 33)	SMBG controls baseline (*n* = 31)	isCGM intervention 6 months (*n* = 33)	SMBG controls 6 months (*n* = 31)	*p*	OR (95% CI) adjusted	*p*
Subjective sleep quality	1 (1, 2)	2 (1, 2)	1 (1, 2)	1 (1, 2)	0.704	1.11 (0.36, 3.40)	0.851
Sleep latency	1 (1, 2)	2 (1, 2)	1 (1, 3)	1 (0, 2)	0.586	0.97 (0.35, 2.67)	0.953
Sleep duration	0 (0, 2)	1 (0, 2)	0 (0, 1)	0 (0, 1)	0.996	0.83 (0.22, 3.09)	0.785
Sleep efficiency	0 (0, 1)	0 (0, 2)	0 (0, 1)	0 (0, 1)	0.573	1.28 (0.37, 4.41)	0.694
Sleep disturbances	1 (1, 2)	1 (1, 2)	1 (1, 2)	1 (1, 1)	0.584	2.41 (0.72, 8.12)	0.154
Use of sleep medications	0 (0, 0)	0 (0, 1)	0 (0, 0)	0 (0, 1)	0.126	0.46 (0.10, 2.12)	0.319
Daytime dysfunction	1 (1, 2)	1 (1, 1)	1 (1, 1)	1 (0, 1)	0.435	2.06 (0.52, 8.15)	0.304
PSQI global score	6 (5, 9)	8 (5, 11)	5 (3, 10)	6 (4, 9)	0.781	0.91 (0.33, 2.52)	0.860

PSQI, Pittsburgh sleep quality index; isCGM, intermittently scanned continuous glucose monitoring; SBMG, self-monitoring blood glucose; OR, odds ratio; CI, confidence interval. PSQI quality domain scores range 0–3; PSQI global score ranges 0–21; categorical data presented as median (25^th^, 75^th^ percentiles); *p* values for unadjusted differences in PSQI domain between the groups from Mann−Whitney *U* tests; OR (95% CI) for isCGM and SMBG groups rating “poor” scores in each domain presented after adjustment for age at 6 months.

## Data Availability

The data are available upon request from the authors.
